# Metastability induced by non-reciprocal adaptive couplings in Kuramoto models

**DOI:** 10.3389/fnetp.2026.1774273

**Published:** 2026-03-26

**Authors:** Sayantan Nag Chowdhury, Hildegard Meyer-Ortmanns

**Affiliations:** 1 School of Science, Constructor University, Bremen, Germany; 2 Complexity Science Hub, Vienna, Austria

**Keywords:** adaptive coupling, Kuramoto model, metastable dynamics, neuronal networks, non-reciprocity, synchronization, network physiology

## Abstract

Non-reciprocal couplings are frequently found in systems out-of-equilibrium such as neuronal networks. Via bifurcation analysis and numerical integration we consider generalized Kuramoto models with non-reciprocal adaptive couplings. The non-reciprocity refers to the type of couplings according to Hebbian or anti-Hebbian rules and to different time scales on which the couplings evolve. The main effect of this specific combination of deterministic dynamics is an induced metastability of anti-phase synchronized clusters of oscillators. The time series exhibit random features but arise from deterministic dynamics. We analyze the metastability as a function of the system parameters, in particular of the size and the network connectivity. Metastable switching is typical for neuronal networks and a characteristic of brain dynamics. The mechanism behind the observed sudden changes in the order parameters is individual oscillators which change their cluster affiliation from time to time, providing “weak ties” between clusters of synchronized oscillators, where an individual oscillator may represent an entire brain area. This mechanism provides an alternative way of inducing metastability in the oscillatory system to switching events as result of heteroclinic dynamics.

## 1 Introduction

To bridge the gap between behavioral and neural views of learning, neural analogues of behavioral modification paradigms have been postulated. An example of such an attempt was D. Hebb’s suggestion in 1949 (Hebb, 1949) that when a cell A repeatedly and persistently takes part in firing another cell B , $A^{\prime }s$ efficiency in firing B is increased. This is a rule for synaptic plasticity. It amounts to an analogue of associative conditioning. It is then typical for neural network models to employ a presynaptic and a postsynaptic correlation rule for altering connectivity as a mathematical representation of Hebb’s postulate. Inspired by Hebb’s seminal work, Rosenblatt proposed the perceptron (Rosenblatt, 1958) to establish a relation between biophysics and psychology. His goal was to predict learning curves from neurological variables and vice versa, neurological variables from learning curves. He proposed a quantitative statistical approach to the organization of cognitive systems. This was in 1958. Already in the seventies and eighties, many adaptive neural network theories were considered in which neuron-like adaptive elements behave as single unit analogues of associative conditioning. In particular, such an adaptive element was proposed in (Sutton and Barto, 1981) that respects facts of animal learning theory. It implements an essential feature of classical conditioning that the element learns to increase its response rate in anticipation of increased stimulation. Later many theoretical studies of learning in neural systems were based on the Hopfield model (Hopfield, 1982) and McCulloch-Pitts units (Hayman, 1999) or “integrate-and-fire” models of neurons, coupled via synapses that accumulate the presynaptic activity into an increasing membrane potential of neurons which fire when a certain threshold is crossed (Clavering Tuckwell, 1988). The synaptic strength there is not fixed.

In a complementary approach, neuronal activity is modeled by periodic oscillators which replace the neurons or entire sub-populations of neurons such as cortical areas. The reason is that rhythmic activity is observed, for example, in central pattern generators, visual or olfactory systems (Gray, 1994). When neural networks are described as networks of coupled oscillators, the role of relative spike timing is played by the individual oscillator phases. It was Winfree (Arthur, 1967) who advanced a reduction of the oscillator dynamics to a pure phase dynamics if the coupling is assumed to be weak, he discovered that a transition to synchronization occurs when the coupling strength exceeds a certain threshold. Kuramoto then proposed the sine interaction term which made the model analytically solvable. This led to the proposal of Kuramoto’s model of phase oscillators (Kuramoto, 2005; Kuramoto, 1984), for a later review see (Acebrón et al., 2005). In its original version, globally coupled Kuramoto oscillators with nonidentical frequencies exhibit a second-order phase transition to a synchronized state as the coupling strength increases above a critical value. Various generalizations of the Kuramoto model have been considered afterwards.

One of the first studies of plasticity and learning in a network of coupled phase oscillators is the work of (Sompolinsky and Kanter, 1986) and somewhat later the work of (Seliger et al., 2002). Plasticity refers to the change in the synaptic weights in the context of neural networks, it is a special form of general adaptation dynamics for which the couplings adapt to the dynamics of the dynamical units assigned to the nodes of the network. Also beyond applications to neural networks, coupling adaptation in oscillatory systems leads to many interesting effects, since the level of description is abstract enough to allow different interpretations. Insights from neural networks with adaptive couplings can be exploited to explain dynamical properties of multi-frequency clusters in power grid networks, described by Kuramoto-Sakaguchi phase oscillators with inertia (Berner et al., 2021a). The fundamental relation between power grid and neural networks is established in (Berner et al., 2021b); according to this relation phase oscillator models with inertia correspond to a particular class of adaptive networks. Modular structure on meso- and macroscales can emerge when adaptively reinforced synchronization competes with a constraint on nodes to establish connections with other units of the network (Gutiérrez et al., 2011). Adaptation is also considered for adaptive multiplex networks, see, for example, the work of (Zhang et al., 2015; Berner et al., 2020; Sawicki et al., 2022). Adaptive coupling can have the interesting effect of inducing collective excitability and self-sustained bursting oscillations in globally coupled populations of non-excitable units (Ciszak et al., 2020). For oscillatory neuronal populations with spike-timing dependent plasticity it is shown in (Popovych et al., 2013) that for an optimal noise level the amount of synaptic coupling gets maximal, leading to noise-induced self-organized synaptic connectivity. Here, the application of noise counteracts desynchronization that would naively be expected as result of noise.

According to the work of (Kasatkin et al., 2017), a multilayer structure and chimera states emerge in a self-organized way if generalized globally coupled Kuramoto models are considered with adaptive couplings. Subnetworks of densely coupled elements form sequentially whose size is hierarchically ordered and which decouple as result of the hierarchical structure. If the global coupling in such networks is replaced by a random sparse structure of connections, chimera states form which retain features of a hierarchical organization, and the set of elements that form coherent groups can be rearranged during the network evolution (Kasatkin and Nekorkin, 2018). When the couplings adapt and have their own dynamics, it amounts to plasticity in relation to neuronal networks. In particular, the couplings may change as a function of the timing between the oscillators. If spike-time dependent plasticity also enters into the natural frequencies of the oscillators, heterogeneous layered clusters with different frequencies show up from homogeneous oscillator populations (Aoki, 2015). In this paper we will consider special cases of the work of (Kasatkin et al., 2017; Kasatkin and Nekorkin, 2018; Aoki, 2015).

More recently, possible biological or neurological mechanisms related to learning, plasticity, and adaptation are reviewed in (Papo and Buldú, 2025). Hebbian learning is one important mechanism of brain plasticity, but Hebbian learning alone would lead to dynamic instability and requires compensatory processes on multiple timescales (Zenke and Gerstner, 2017). Various mechanisms are listed in (Papo and Buldú, 2025) to achieve dynamic stability. It seems still to be relatively unknown at which spatial and temporal scales Hebbian, homeostatic, and other plasticity mechanisms interact, and which functional role they play. In this work, we will consider two plasticity mechanisms, Hebbian and anti-Hebbian, acting at quite different time scales, but this choice is not motivated by neurological experiments, it serves to explore the effect of non-reciprocity.

At another and very different frontier, non-reciprocity is topical in systems out-of-equilibrium, since the validity of Newton’s third law (for every action is an equal and opposite reaction) is the exception rather than the rule in these systems. Arguably the most plausible example is from social systems where a relation of friendship holds in one direction but more enmity than friendship in the opposite direction. Populations with conformist and contrarian members are considered in (Hong and Strogatz, 2011). Non-reciprocity is also found in active matter (Lavergne et al., 2019; Uchida and Golestanian, 2010; N et al., 2010), metamaterials (Brandenbourger et al., 2019; Miri and Alu, 2019), or in relation to game theory (Nag Chowdhury et al., 2020). Occasionally it is discussed in neuronal networks (Sompolinsky and Kanter, 1986; Montbrió et al., 2018). In (Sompolinsky and Kanter, 1986), asymmetric networks are considered, where only part of the synaptic connections between pairs of neurons are non-reciprocal and have slow dynamic response with the effect that temporal association becomes possible as recall of time sequences and cycles of patterns. The work of (Montbrió et al., 2018) introduces two populations of excitatory and inhibitory populations in a Kuramoto model with the non-reciprocal feature that each oscillator of the excitatory (inhibitory) population exerts a positive (negative) influence on each oscillator of the inhibitory (excitatory) population, respectively. This way, the Kuramoto model accounts for the onset of excitatory/inhibitory-based neuronal rhythms.

Particularly in view of collective behavior, non-reciprocity can have strong implications on many-body systems. It may lead to time-dependent phases in which spontaneously broken continuous symmetries are dynamically restored (Fruchart et al., 2021). In view of general manifestations of non-reciprocity, we consider systems with self-organizing structures, of which synchronization is a prototypical form. More specifically, this paper is on non-reciprocity in adaptive networks and its impact on synchronization patterns. The dynamics of non-reciprocal adaptation may be realized in many ways, via the type of favored interaction (alignment or anti-alignment, Hebbian or anti-Hebbian rules), via the choice of time scales, via feedback as in (Sompolinsky and Kanter, 1986) or frequency adaptation to name some of them.

In this paper, we focus on generalized Kuramoto models with both adaptive and non-reciprocal couplings. When we combine Hebbian and anti-Hebbian adaptation rules for the couplings, acting on quite different time scales, we observe switching dynamics between metastable states, characterized by anti-phase cluster synchronization (exceptionally also full synchronization) unless the choice of parameters leads to incoherent oscillations. Emerging metastability is the main novel feature of our results. Therefore, the results may be relevant to neural network applications, since many experimental hints exist that typical states in the brain are metastable (Tognoli and Scott Kelso, 2014). The structure of these metastable states is reflected in functional neuroimaging experiments. In particular cognitive phenomena rely on transient dynamics such as working memory and decision making (Rabinovich et al., 2008; Rabinovich et al., 2011). The idea is that brain activity is organized in spatiotemporal patterns through transient metastable states (Scott Kelso, 1995; Friston, 1997). For a long time the search was for a dynamic framework that describes transient reproducible and metastable dynamics. One such framework is provided by heteroclinic dynamics, for a recent review see (Meyer-Ortmanns, 2023). For a review on metastable dynamics see, for example, (Rossi et al., 2025).

In this paper, we demonstrate how -differently from heteroclinic dynamics a set of deterministic equations can also lead to switching dynamics, here induced by a fast change between attractive and repulsive couplings in half of the node connections, and a slow change between both types in the other half. In detail we consider an all-to-all coupling topology, but we also present results for a random network, for which we vary the density of connectivity. We briefly consider effects of non-reciprocal adaptivity when also the natural frequencies become coupling dependent.

The paper is organized as follows: In Section 2 we introduce different versions of the model. Section 3 presents the results in terms of synchronization patterns as function of the model parameters, in particular of the time scales, the system size, and the grid topology. Section 4 contains our conclusions. In the Supplementary Material 1 we provide an analytical understanding of the increasing instability with the system size.

## 2 The model

We consider a population of N≥2 phase oscillators, each described by its phase $\theta_{i}\left(t\right)\in \left.\left[0,2\pi \right.\right)$ , whose dynamics evolve according to a modified Kuramoto model with adaptive non-reciprocal couplings. The evolution equation for the i -th oscillator is given by

$${\dot{\theta }}_{i}=\omega_{i}+\frac{1}{N}\overset{N}{\underset{j=1}{\sum }}k_{ij}\sin\left(\theta_{j}-\theta_{i}\right)+\xi_{i}\left(t\right),$$ (1)

where $\omega_{i}$ denotes the intrinsic natural frequency, and $k_{ij}$ is the time-dependent coupling strength, with i,j=1,…,N . The coupling is all-to-all and directed, and the matrix elements $k=\left[k_{ij}\right]$ evolve as

$${\dot{k}}_{ij}=\left\{\begin{array}{cc} -\varepsilon_{1}\left[k_{ij}+\sin\left(\theta_{i}-\theta_{j}+\beta_{1}\right)\right], & \text{for }i<j,\\ -\varepsilon_{2}\left[k_{ij}+\sin\left(\theta_{i}-\theta_{j}+\beta_{2}\right)\right], & \text{for }i>j,\\ 0, & \text{for }i=j. \end{array}\right.$$ (2)

We choose $\beta_{1}=-\frac{\pi }{2}$ and $\beta_{2}=+\frac{\pi }{2}$ . The parameters $\varepsilon_{1},\varepsilon_{2}>0$ set the different time scales for the adaptation rates for the upper and lower triangular parts of the matrix. The diagonal elements of the coupling matrix, $k_{ii}$ , are set to zero to avoid self-coupling. This means that for connections from a lower-index oscillator i to a higher-index oscillator j , the coupling tends to adjust with a phase lag of −π/2 (Hebbian plasticity rule, where the weights for a pair of in-phase (or anti-phase) oscillators will increase (or decrease), respectively) see, for example, (Aoki, 2015). For connections in the opposite direction, the adjustment follows a phase lead of +π/2 (anti-Hebbian rule), discussed, for example, in (Kasatkin et al., 2017). For simplicity the upper-triangular (lower-triangular) couplings $k_{ij}$ with i<j (i>j) are termed the upper (lower) couplings in the following. The term $\xi_{i}\left(t\right)$ represents additive Gaussian white noise of intensity σ :

$$\langle \xi_{i}\left(t\right)\rangle =0,\langle \xi_{i}\left(t\right)\xi_{j}\left(t^{\prime }\right)\rangle =\sigma^{2}\delta_{ij}\delta \left(t-t^{\prime }\right).$$

Throughout the paper we will keep the slow (fast) time scale associated with the anti-Hebbian (Hebbian) rule, respectively, since otherwise we observe only incoherent oscillations. Our motivation for this choice does not directly come from experiments, it is to test the effect of non-reciprocity when it is implemented in the adaptation dynamics by the type and the time scales of adaptation. Moreover, for $\epsilon_{1}=\epsilon_{2}$ it allows a comparison to the results of the work of (Berner et al., 2019; Kasatkin et al., 2017; Kasatkin and Nekorkin, 2018; Aoki, 2015) for special choices of their parameters. For example, when the phase dynamics includes an additional parameter α to simulate a phase lag of the interaction, we set it to zero unless otherwise stated. In previous studies of (Berner et al., 2023)- (Wang et al., 2020), the adaptation rules were chosen symmetric with $\varepsilon_{1}=\varepsilon_{2}>0$ . In these cases, if the phase shift was $\beta_{1}=\beta_{2}=+\pi /2$ , the system typically remained incoherent (Kasatkin et al., 2017), while if the phase shift was $\beta_{1}=\beta_{2}=-\pi /2$ , two anti-phase clusters could form (Kasatkin et al., 2017).

As a stochastic extension of the standard Euler method, Equations 1, 2 are integrated numerically using the Euler–Maruyama scheme with a fixed time step Δt=0.01 and $\theta_{i}\mapsto \theta_{i}\;\mathrm{mod}\;2\pi$ .

The initial phases $\theta_{i}\left(0\right)$ are sampled uniformly from $\left.\left[0,2\pi \right.\right)$ , while (unless stated otherwise) the initial couplings $k_{ij}\left(0\right)$ are drawn from a uniform distribution in [−1,1] with $k_{ii}\left(0\right)=0$ . The natural frequencies $\omega_{i}$ are in most cases set to zero, for some test runs (not presented) we sampled them from a normal distribution with a fixed mean $\omega_{\text{mean}}$ and a fixed standard deviation $\omega_{\text{std}}$ .

The degree of phase coherence is quantified using two complex quantities, the first Kuramoto order parameter R (Kuramoto, 1984; Acebrón et al., 2005; Daido, 1992) and the second Kuramoto order parameter $R_{2}$ (Daido, 1992):

$$Re^{i\psi }=\frac{1}{N}\overset{N}{\underset{j=1}{\sum }}e^{i\theta_{j}},\;R_{2}e^{i\psi_{2}}=\frac{1}{N}\overset{N}{\underset{j=1}{\sum }}e^{2i\theta_{j}},$$

where R∈[0,1] measures the global synchronization level, and $R_{2}\in \left[0,1\right]$ captures the presence of two anti-phase clusters (Berner et al., 2019). It is particularly useful to characterize our synchronization patterns. The limiting cases are as follows: R≈1 and $R_{2}\approx 1$ for complete in-phase synchronization; R≈0 and $R_{2}\approx 1$ for two anti-phase clusters; and both R and $R_{2}$ small for incoherent dynamics.

After an initial transient, the long-time averaged order parameters ⟨R⟩ and $\left\langle R_{2}\right\rangle$ are computed over a sufficiently large portion of the simulation succeeding the transient. Unless otherwise specified, we simulate the system for a total of $T_{\mathrm{total}}=1.8\times 10^{6}$ time steps. To eliminate initial transient behaviors, we discard the first 30% of the simulation (approx. $5.4\times 10^{5}$ steps) before computing the long-time averaged order parameters ⟨R⟩ and $\left\langle R_{2}\right\rangle$ . As we will see, for our results it is sufficient to classify the collective states as follows: if ⟨R⟩ and $\left\langle R_{2}\right\rangle$ both are greater than a threshold $R_{\text{th}}$ , the system is in the in-phase synchronized state. If ⟨R⟩ is less than or equal to $R_{\text{th}}$ , and $\left\langle R_{2}\right\rangle$ is greater than $R_{\text{th}}$ , the system is in the two anti-phase clusters state. In all other cases, the system is considered incoherent. For the threshold we have chosen $R_{\text{th}}=0.8$ , though usually for anti-phase synchronized clusters $R_{2}>0.8$ . The threshold of $R_{th}=0.8$ acts as a conservative lower bound (Rodrigues et al., 2016; Arenas et al., 2008). In the metastable states that we observe, the synchronized clusters are typically tightly locked, yielding $R_{2}$ values well above 0.9. A threshold of 0.8 reliably separates these coherent states from incoherent regimes while allowing for small fluctuations induced by the adaptive, non-reciprocal coupling dynamics.

## 3 Results

We start with a discussion of the typical stationary states and argue for the choice of parameters which we keep throughout this paper. Next we analyze features of randomness in spite of deterministic dynamics, the dependencies on the initial conditions, the system size, and the effect of disorder. In the last two subsections we consider different versions of the generalized Kuramoto model.

### 3.1 Stationary states and choice of parameters

To visualize the dependence of collective states on the adaptation rates $\left(\varepsilon_{1},\varepsilon_{2}\right)$ , we perform numerical simulations of the coupled system (1)-(2) with N=10 and N=20 oscillators over multiple realizations.

**FIGURE 1:**
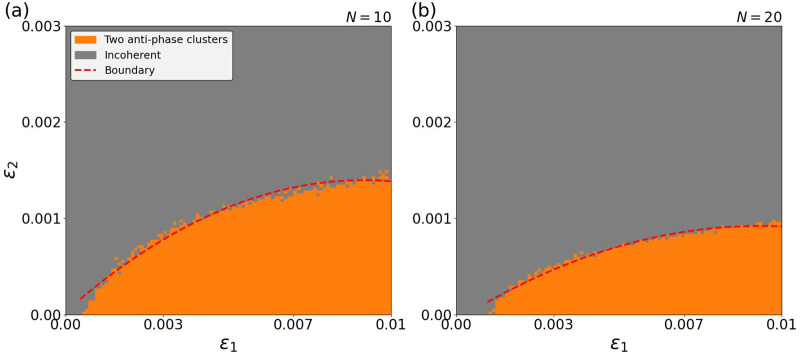
Categorical heatmap of collective states as a function of $\left(\varepsilon_{1},\varepsilon_{2}\right)$ for **(a)** N=10 oscillators and **(b)** N=20 oscillators, averaged over 200 independent realizations. For a specific choice of $\varepsilon_{1},\varepsilon_{2}$ colors indicate the state to which the system converges: orange for two anti-phase clusters, and gray for incoherent states; $\varepsilon_{1}$ is varying within [0, 0.01] and $\varepsilon_{2}$ is varying within [0, 0.003] with a step length of 0.0001. The red dashed curve serves to guide the eyes and roughly marks the transition boundary between two anti-phase clusters and incoherent states via a quadratic least square fit.

The simulations are carried out in the noise-free case with identical intrinsic frequencies $\omega_{i}=0$ for all oscillators i=1,…,N . To track the long-time dynamics, the order parameters, R and $R_{2}$ , are computed and averaged over the last sufficiently long time window. Each parameter pair $\left(\varepsilon_{1},\varepsilon_{2}\right)$ is simulated for 200 independent realizations, and the resulting mean order parameters ⟨R⟩ and $\left\langle R_{2}\right\rangle$ are used to classify the collective state. Specifically for the construction of these phase diagrams, where covering the parameter space with high resolution requires massive computational throughput (200 realizations per grid point), we used a shorter simulation time of $4\times 10^{4}$ steps. The order parameters were averaged over the final 1000 steps after the system had relaxed into its attractor. We verified that this duration is sufficient for the collective state (two anti-phase clusters vs. incoherent) to stabilize. The resulting categorical heatmap is shown in Figure 1. Each pixel corresponds to an $\left(\varepsilon_{1},\varepsilon_{2}\right)$ pair, color-coded by the observed collective state. Since $\varepsilon_{2}>0$ corresponds to the anti-Hebbian coupling, it tends to disrupt phase coherence, and sufficiently large values of $\varepsilon_{2}$ generally lead to incoherent dynamics. In contrast, when $\varepsilon_{1}\gg \varepsilon_{2}>0$ , the upper-triangular Hebbian interactions dominate, promoting the formation of two anti-phase clusters for most parameter realizations. For ε -values in the vicinity of the red dashed line it depends on the random initial conditions in which state the system ends up, therefore the boundary is a bit rough. Figure 1a corresponds to a system of N=10 oscillators, while Figure 1b is for N=20.

**FIGURE 2:**
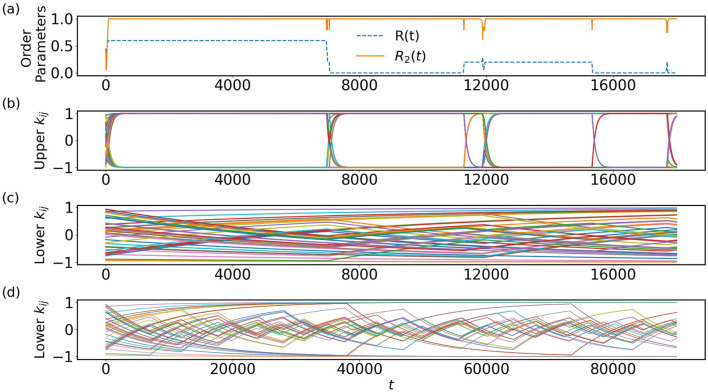
**(a)** Time series of the first- and second-order Kuramoto order parameters R(t) (dashed line) and $R_{2}\left(t\right)$ (solid line) for $\varepsilon_{1}=0.01$ and $\varepsilon_{2}=0.0001$ with N=10 . After initial convergence to $R_{2}\approx 1$ , occasional deviations occur, visible in spikes, which subsequently return to the same state. **(b,c)** Upper part of the couplings $k_{ij}$ (for i<j ) and lower part of $k_{ij}$ (for i>j ). As soon as $R_{2}$ deviates from 1, a few oscillators switch clusters, which leads to changes in the slopes of the corresponding $k_{ij}$ values. Since $\varepsilon_{1}=0.01$ is significantly larger than $\varepsilon_{2}=0.0001$ , the upper $k_{ij}$ values quickly reach their equilibrium values of ±1 , whereas the lower $k_{ij}$ values adjust more slowly. **(d)** Extended time view of **(c)** to see some structure in the evolution of the lower couplings.

To examine the dynamics in detail, we simulate the system (1)-(2) with N=10 oscillators, noise intensity σ=0 and intrinsic frequencies $\omega_{i}=0\;\forall i$ . We fix the coupling parameters at $\varepsilon_{1}=0.01$ and $\varepsilon_{2}=0.0001$ , a regime where two anti-phase clusters are expected from Figure 1a. While one might anticipate that $R_{2}$ would converge to one after an initial transient, we observe metastable behavior: $R_{2}$ initially approaches 1, then occasionally deviates from this value, only to return to $R_{2}\approx 1$ after a few iterations. We observe the same recurrent deviations of $R_{2}$ as shown in Figure 2a) also over a total integration time of up to $9\times 10^{7}$ steps.

As seen in Figure 2a, also the standard Kuramoto order parameter R shows metastable behavior. Depending on the degree of synchronization, the value of R after a spike may be different, and whenever $R_{2}$ is perturbed, R experiences a corresponding disturbance. During $R_{2}=1$ , the two anti-phase clusters remain well separated by a phase difference of π , although the number of oscillators in each cluster may vary temporarily during the disturbances of $R_{2}$ . After a spike in $R_{2}$ , the system returns to a state with $R_{2}\approx 1$ , preserving the two-cluster structure with π phase difference. Thus, a spike in the order parameter $R_{2}$ indicates a short deviation from the organization into two anti-phase synchronized clusters which obviously corresponds to an unstable solution. It is instructive to plot the evolution of the asymmetric coupling matrix $k_{ij}$ in Figures 2b,c. In panel (b), we observe the time evolution of the upper part $k_{ij}$ , that is, for i<j . As the system evolves, some of the couplings settle temporarily at +1 , while others stabilize temporarily at −1 . However, when $R_{2}$ deviates from 1 , there are switches in some of the $k_{ij}$ values from +1 to −1 or vice versa, indicating a temporary departure from the anti-phase synchronized two-cluster state. At the same time, some of the lower couplings $k_{ij}$ , that is, for i>j also undergo slope changes, as shown in panel (c), but evolving on a much slower time scale. Note that the transient dynamics of the order parameters R and $R_{2}$ appear slightly faster even than those of the upper couplings $k_{ij}$ (Figures 2a,b). This is due to the timescale separation explicitly built into the model: the phases $\theta_{i}$ evolve on a timescale of O(1) (Equation 1), whereas the upper part of the couplings evolve with rate $\varepsilon_{1}=0.01$ . Consequently, the oscillators can phase-lock and stabilize R and $R_{2}$ relatively quickly, while the coupling weights require some integration time to saturate to their equilibrium values. Panel (d) displays the evolution of the lower couplings on a long time scale, where it becomes more clearly visible that not all couplings are affected by a switch of $R_{2}$ . Here one may wonder whether some periodic structure is seen on long time scales.

Metastability as an essential novel feature. Metastability is the essential novel feature that we observe in these combinations of non-reciprocal couplings with respect to their sign and time scales. It is absent in the reciprocal case (Kasatkin et al., 2017). Even, when $\varepsilon_{1}\gg \varepsilon_{2}$ with $\varepsilon_{1}=0.01$ , $\varepsilon_{2}=0.0001$ , but $\beta_{1}=\beta_{2}$ , the system does not exhibit metastability. In this case, when $\beta_{1}=\beta_{2}=-\frac{\pi }{2}$ , two stable anti-phase clusters emerge, while for $\beta_{1}=\beta_{2}=+\frac{\pi }{2}$ , oscillations are only incoherent. Metastability, on the other hand, is observed in our system when there is sufficient asymmetry in the adaptive coupling dynamics, specifically when $\varepsilon_{1}\gg \varepsilon_{2}>0$ , as shown in the orange-colored region of Figure 1 as well as $\beta_{1}\neq \beta_{2}$ . The slow dynamics acts as a perturbation on the fast dynamics; for the fast dynamics alone we would see a kind of dynamic realization of Harary’s theorem (Harary, 1960). According to that, the set of all points of a balanced signed graph can be partitioned into two disjoint sets such that each positive line joins two points of the same subset and each negative line joins two points from different subsets, the lines are undirected. In our case, we have directed links with different dynamics assigned to both directions; but as long as the lower couplings are neglected which act in opposite link direction to the upper ones, what dynamically matters is which dynamics is assigned to the lines: these are attractive upper couplings, corresponding to positive lines, and realized as zero phase difference within the same cluster, as well as repulsive upper couplings, corresponding to negative lines, and realized as phase difference of π between members of different clusters. The two anti-phase synchronized clusters are then slightly perturbed by the slow dynamics of lower couplings which is manifest in some oscillators changing their cluster affiliation from time to time.

**FIGURE 3:**
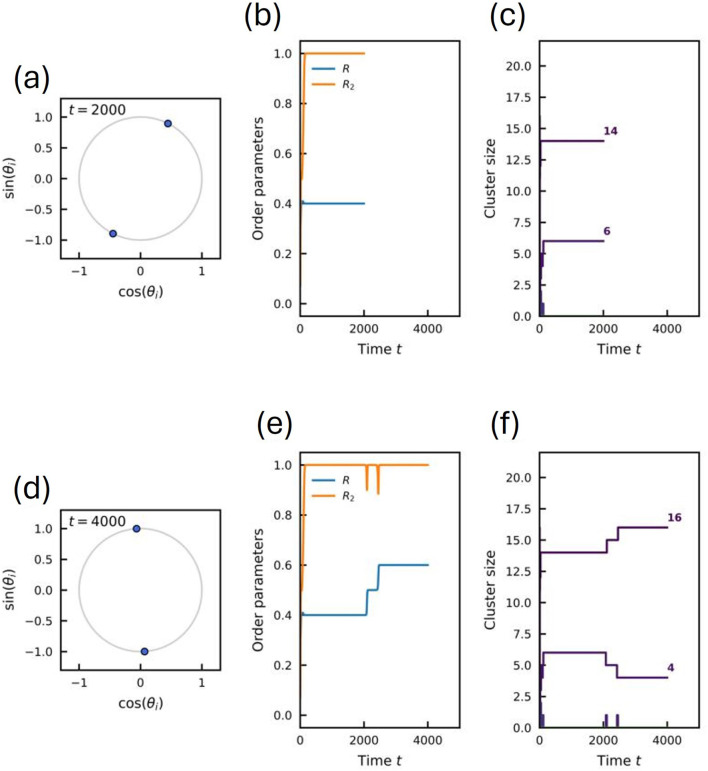
Snapshots of the system state before and after a switching event for N=20 . **(a–c)** State at t=2000 (before the switch). **(a)** Instantaneous phases on the unit circle showing two well-separated clusters. **(b)** Time evolution of the order parameters R and $R_{2}$ . **(c)** Time evolution of the cluster sizes, showing a partition into 14 and six oscillators. **(d–f)** State at t=4000 (after the switch). **(d)** Instantaneous phases. **(e)** Evolution of order parameters; note the jump in R and $R_{2}$ coinciding with the switch. **(f)** Cluster sizes reorganize into a partition of 16 and 4 oscillators.

Switching the cluster affiliation. Numerically we confirm that occasionally some oscillators switch groups, causing $R_{2}$ to deviate from 1. After a few steps, these oscillators return to a possibly slightly different anti-phase cluster configuration, and $R_{2}$ revisits the value of 1. The number of oscillators in each cluster may change with each deviation of $R_{2}$ from 1. Here, we include four videos (Sayantan Nag Chowdhury, 2025) for different system sizes, N=20 and N=200 , demonstrating these features, in particular how the group sizes may change with each spike in $R_{2}$ . In Figure 3, we include snapshots of these movies before and after a spike in $R_{2}$ .

What causes the changes in the slope of fast and slowly evolving couplings and which couplings are affected? Let us consider the simplest case, where a single oscillator l changes its cluster affiliation. What happens to the couplings $k_{lk}$ in which this oscillator is involved? From the evolution of the coupling strengths $k_{ij}$ given in Equation 2 it is seen that two oscillators with indices l and k , chosen from the N oscillators, which initially belong to the same cluster at time $t=t_{1}$ in an anti-phase cluster configuration with $R_{2}=1$ evolve as

$${\dot{k}}_{lk}=-\varepsilon_{1}\left(k_{lk}-1\right),\;\;\text{for }l<k,\;{\dot{k}}_{lk}=-\varepsilon_{2}\left(k_{lk}+1\right),\;\;\text{for }l>k.$$

Since $k_{lk}\in \left[-1,1\right]$ and $\varepsilon_{1},\varepsilon_{2}>0$ , these relations imply ${\dot{k}}_{lk}\geq 0,\;\;\text{for }l<k$ , and ${\dot{k}}_{lk}\leq 0,\;\;\text{for }l>k$ . Thus, when oscillators l and k are in the same cluster, the upper–triangle elements of the coupling matrix (l<k) increase toward +1 , while the lower–triangle elements (l>k) decrease toward −1 . Now suppose that at time $t=t_{2}>t_{1}$ , oscillator l switches its affiliation to the opposite cluster, so that now $\theta_{l}-\theta_{k}=\pi$ . In this case, the coupling dynamics becomes

$${\dot{k}}_{lk}=-\varepsilon_{1}\left(k_{lk}+1\right),\;\;\text{for }l<k,\;{\dot{k}}_{lk}=-\varepsilon_{2}\left(k_{lk}-1\right),\;\;\text{for }l>k.$$

and, because $k_{lk}\in \left[-1,1\right]$ , we obtain ${\dot{k}}_{lk}\leq 0,\text{for }l<k$ , and ${\dot{k}}_{lk}\geq 0,\text{for }l>k$ .

In conclusion, this analysis shows that as long as two oscillators are in the same cluster, which is a metastable configuration, the couplings $k_{lk}$ evolve toward the saddle-equilibrium ${\dot{k}}_{lk}=0$ with either positive or negative slope. However, when one of the oscillators switches to the opposite cluster, the sign of the phase difference changes by π , and the slope of evolution of $k_{lk}$ reverses. Due to the difference in time scales this happens fast for upper couplings and very slowly for lower ones, so slowly that the lower couplings do not reach the equilibrium values of ${\dot{k}}_{lk}=0$ before the next switch happens.

Numerically and visible in the attached movies we observe that depending on the system size, for small sizes N=10 or N=20 , it is a single or a few oscillators that change their cluster affiliation at the spikes of $R_{2}$ , for larger sizes these are more. What is typical in the movies is the splitting into two clusters of similar size as well as the fact that only a few oscillators change their affiliation. This reminds is reminiscent to “weak ties” in social systems, where a few members of a community are weakly bound and willing to switch their community (of shared opinions, for example,), while the majority is strongly bound, and the weak ties are of beneficial impact on the system, as analyzed by Granovetter (Mark, 1973). The reason why oscillators change their cluster affiliation is determined by the attractor landscape and the saddles generated by the dynamics of Equations 1, 2 that we analyze in Section 3.4. In brain dynamics this would correspond to oscillatory units which easily switch the group with which they synchronize, where a single unit may represent a population of neurons.

### 3.2 Features of randomness

The time evolution of the order parameters appears to be generated by a stochastic source in spite of the fully deterministic dynamics of Equations 1, 2. The events when one or more oscillators change their cluster affiliation look randomly distributed. The only source of randomness is the quenched distribution of initial phases and couplings which determine the initial distance of the phases from the saddle equilibria and therefore their trajectories through attractor space. Actually, on an intermediate time scale of the order of the interval between two switching events, the distance from the next saddle is essential to predict which oscillator escapes first in a next switching event. Assuming that there is a switch at time t of one or a few oscillators and that we know the instantaneous phase velocities ${\dot{\theta }}_{i}$ at an earlier time $t_{\mathrm{snap}}=t-\tau_{\mathrm{win}}$ from a snapshot at that time, we approximate the estimated time of escape (ETE) for each oscillator i as

$$\eta_{i}=\frac{\left|\theta_{B}-\theta_{i}\left(t_{\text{snap}}\right)\right|}{{\dot{\theta }}_{i}\left(t_{\text{snap}}\right)\;dt}.$$

Here $\theta_{B}$ is chosen as π . This choice is motivated by the circular topology of the phase space: π marks the point of maximum phase separation from the origin (0 or 2π ), and crossing it signals a definitive change in cluster membership, since we have two anti-phase synchronized clusters. ${\dot{\theta }}_{i}\left(t_{\text{snap}}\right)\;dt$ represents the phase displacement per simulation increment at the time of the snapshot, so the ratio on the right-hand side gives the time in units of simulation steps it takes oscillator i to overcome distance $\theta_{B}-\theta_{i}\left(t_{\text{snap}}\right)$ . This time is minimal for the oscillator $i^{*}$ which is the first to change its cluster affiliation.

$$i^{*}=\arg\min_{i}\left(\eta_{i}\right).$$

Note that the snapshot of velocities ${\dot{\theta }}_{i}\left(t_{\text{snap}}\right)$ depends on the upper and lower couplings which coevolve with the phases. However, our previous figures for $k_{ij}$ show that within two switching events upper couplings are almost constant, as they converge fast to their equilibrium values, and lower couplings are so slow that their change may be neglected. Moreover, the distance is assumed to increase linearly with time. Nevertheless this approximation works quite well to predict the dynamics after a switch including an identification which oscillator will switch first.

Before we discuss the concrete results, some remarks are in order about the choice of $\tau_{\text{win}}$ , $\tau_{\text{win}}=200$ turns out to be a good choice, given the other parameters. If $\tau_{\text{win}}$ is chosen too large, the co-evolution of couplings can no longer be neglected, oscillators whose ETE exceeds a cutoff value $\eta_{\max}=500$ steps are excluded from consideration. If $\tau_{\text{win}}$ is too short, there is no prediction. If more than one oscillator switches its cluster affiliation, we group individual switches that happen very close together into a single collective event. If several oscillators overcome the distance of π within 50 simulation steps of each other, they are evaluated as a single event. For entire groups changing their affiliation, we predict the membership of the switching cluster P by including any oscillator j whose ETE lies within a temporal margin Δη of the “leading oscillator” i : $P=\left\{j|\eta_{j}\leq \eta_{i^{*}}+\Delta \eta \right\}$ . This margin accounts for the finite temporal spread of collective switching events and is set to Δη=150 steps in our simulations, based on the typical duration of observed group switching.

**FIGURE 4:**
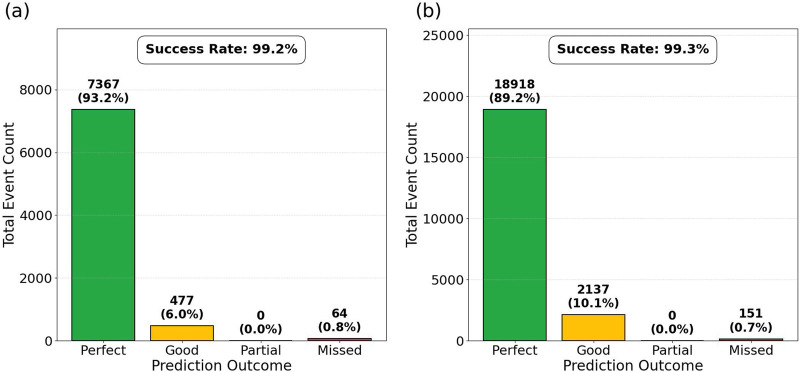
Snapshot-based prediction performance across 1,000 random realizations for **(a)** N=6 and **(b)** N=10 . The bars represent the total count of switching events categorized by prediction accuracy. The percentage shown above each bar indicates the frequency of that outcome relative to the total number of detected events. The overall success rate (combined *Perfect* and *Good* categories) remains remarkably stable as the system size increases.

By comparing the predicted set P with the actual set of oscillators A that overcome a distance of π at time t , predictions are classified into four categories: Perfect: P=A . The predicted group exactly matches the actual switchers. Good: A ⊂ P . All actual switchers were identified, but additional oscillators were incorrectly included. Partial: P∩A≠∅ but A⊄P . Some switchers were correctly identified, while others were missed. Missed: P∩A=∅ . No actual switchers were identified. To skip initial transient behavior, only switching events occurring after $t>0.3\;T_{\text{total}}$ are included from overall $T_{\text{total}}=1.8\times 10^{6}$ steps. The performance of our snapshot-based prediction is summarized in Figure 4 for both N=6 and N=10 nodes. For the N=6 configuration (Figure 4a), the framework achieved an overall success rate of 99.2% , with 93.2% of events classified as *Perfect*. This indicates that the instantaneous state 200 steps prior to a switch contains nearly all the information required to identify the switching cluster. As the system size increases to N=10 (Figure 4b), the predictive robustness is maintained with a success rate of 99.3% . Interestingly, while the proportion of *Perfect* predictions remains high at 89.2% , there is a slight increase in the *Good* category ( 10.1% compared to 6.0% in the N=6 case). This shift suggests that in larger coupled systems, the “lead” oscillator exerts a stronger influence on its neighbors, occasionally causing the inclusion of ‘follower’ oscillators in the predicted set P that do not quite reach $\theta_{B}$ within the expected window. The zero occurrence of *Partial* predictions across all realizations demonstrates that the temporal margin Δη=150 is very effective at capturing events where more oscillators are involved. Furthermore, the consistently low rate of *Missed* events (<1%) confirms that cluster transitions in the adaptive Kuramoto model are predictable within an intermediate time scale. Our approach relies exclusively on a single temporal snapshot of the system state.

**FIGURE 5:**
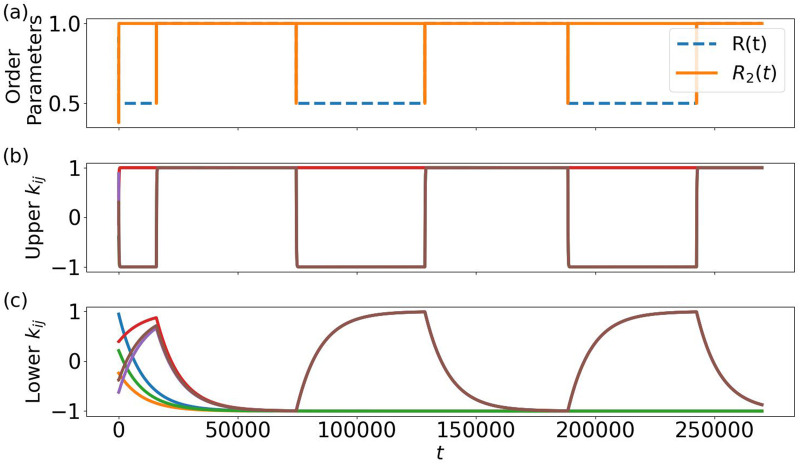
Periodic and synchronized evolution not only of phases **(a)**, but also of upper **(b)** and lower **(c)** couplings into one or two clusters for a network of N=4 oscillators with only one saddle. Other parameters as in Figure 2.

What cannot be predicted is the new partition into two clusters after the switching event and the dynamics on a long-time scale. One might be interested in the probability that a given set of oscillator phases finds the same couplings (actually coupling conditions for their own evolution) after some possibly long time, as the evolution -locally in time-looks random. The question for finding repeatedly the same conditions is the question for long-time periodicity. Such long periods in the Kuramoto order parameter from short period oscillations have been observed in (Labavić and Meyer-Ortmanns, 2017). For larger networks we cannot answer this question, but for a system of N=4 oscillators, the six upper couplings and the six lower couplings synchronize into one or two clusters, as well as the phases, both for N=4 and N=3 the system has only one saddle point and the evolution of all couplings is strictly periodic, see Figure 5.

In summary, on intermediate time scales it is the momentary distance from π of the phase position and the phase velocity at that instant of time, which allow a prediction of which oscillator (group of oscillators) will escape next, knowing that there is an escape event. As soon as this distance is overcome, we can be sure to be in the attraction regime of the other cluster. It is the random initial conditions which determine the initial phase position and lead to an ensemble of time evolutions unless identical initial conditions are chosen via the same seed. Given a set of fixed initial conditions, the waiting time distribution between two switches of the order parameter $R_{2}$ looks random on longer time scales, unless it happens to become periodic. We address this irregularity to the complex attractor landscape that allows the escape from saddles and determines the entrance to the attraction regimes of new saddles. The entrance location determines the dwell time in the vicinity of the new saddles.

### 3.3 Choice of initial conditions and natural frequencies

Before we discuss the stability properties as a function of the system size, some remarks are in order about the choice of initial conditions and the values of the natural frequencies. If we keep all other parameters the same and set the intrinsic frequencies $\omega_{i}$ to a homogeneous nonzero value, e.g., $\omega_{i}=3$ , we observe that the phases $\theta_{i}$ oscillate over time with constant phase difference of π between the temporarily forming two anti-phase synchronized clusters, otherwise the main features of metastability remain qualitatively the same as for $\omega_{i}=0$ . This is expected as we can go to a co-rotating frame and transform the system to zero frequency.

**FIGURE 6:**
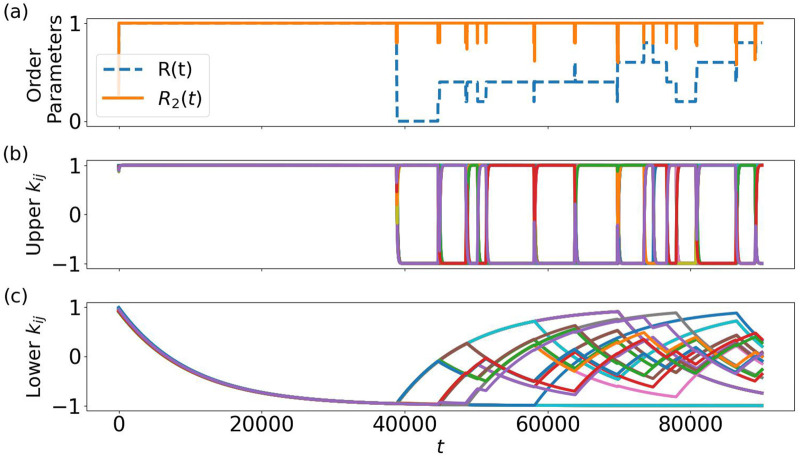
Dependence on the initial conditions of order parameters **(a)**, upper **(b)** and lower **(c)** couplings, here both chosen from a distribution of $k_{ij}$ close to +1 . Note that the first long transient corresponds to a fully synchronized solution, observed very rarely as transient.

If we choose uniformly distributed initial configurations for upper and lower couplings very close to 1, this choice results in some delay of the onset of metastable dynamics, where for the upper couplings it is the fully synchronized state that remains stable until metastability sets in, see Figure 6.

### 3.4 Metastability as a function of the system size

**FIGURE 7:**
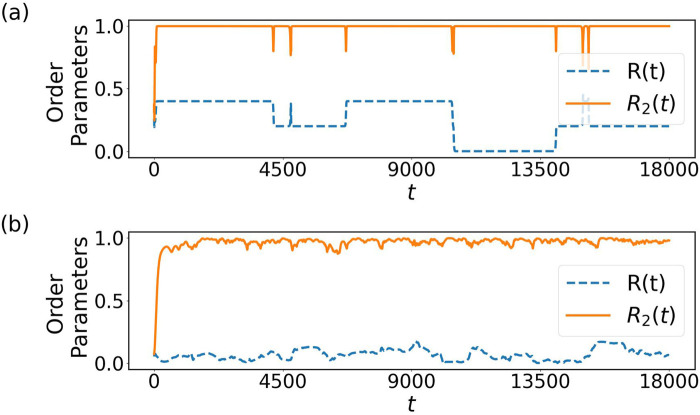
Dependence on the system size for **(a)** N=10 and **(b)** N=200 oscillators with the same parameters as in Figure 2. Compared to the smaller system (N=10) , the fluctuations in $R_{2}$ are more frequent, indicating a larger instability, but less pronounced in size, that is, smaller deviations from the anti-phase synchronized clusters for N=200 . Still, all values of $R_{2}$ remain close to one, corresponding to an organization into two anti-phase clusters.

**FIGURE 8:**
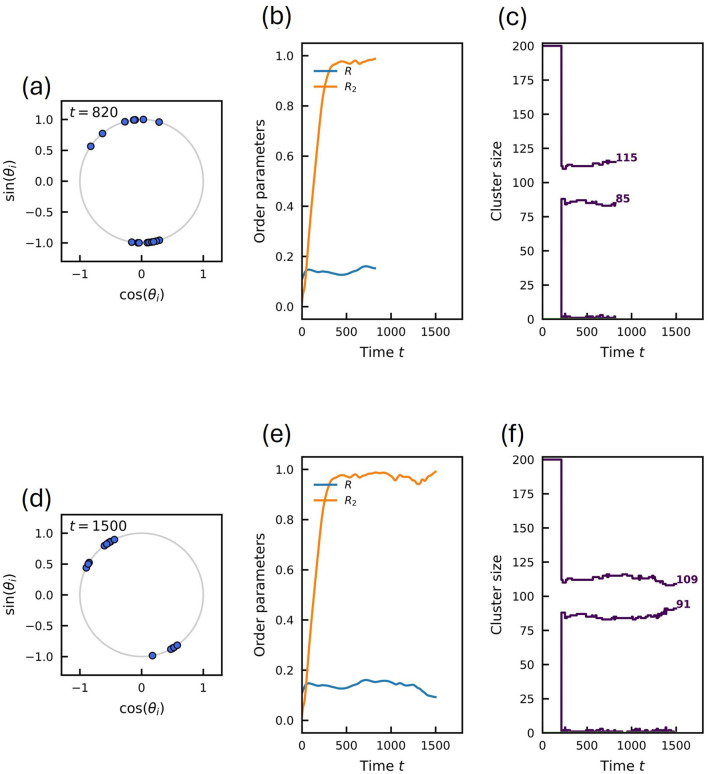
Snapshots of the system state before and after a switching event for the larger system size N=200 . **(a–c)** State at t=820 (before the switch). **(a)** Instantaneous phases on the unit circle; note that the two clusters are less perfectly synchronized than in the N=20 case. **(b)** Time evolution of order parameters. **(c)** Cluster sizes showing a partition of 115 and 85 oscillators. **(d–f)** State at t=1500 (after the switch). **(d)** Instantaneous phases. **(e)** Evolution of order parameters. **(f)** Cluster sizes have shifted to a partition of 109 and 91 oscillators. Compared to N=20 , the fluctuations in cluster size are more frequent, involving multiple oscillators switching affiliation.

In Figure 7 we compare the evolution of the order parameters as a function of the system size for N=10 in Figure 7a and N=200 in Figure 7b. For N=200 the distinction between temporarily stable configurations interrupted by short excursions to other clusters becomes less pronounced. The movie of (Sayantan Nag Chowdhury, 2025) for N=200 shows the evolution of phases, snapshots are given in Figure 8 before and after a spike in $R_{2}$ . We still see remnants of an organization into two anti-phase synchronized clusters, where on short time scales always some oscillators escape and return to their cluster, more frequent are the events where the escape succeeds over a distance π in the phases. At intermediate times also configurations with several coexisting clusters are forming, leading to stronger spikes in $R_{2}$ .

To understand more quantitatively the increasing instability with the system size, we summarize the stability analysis in the following. It is detailed in the Supplementary Data Sheet 1. For the fully connected Kuramoto model with $\omega_{i}=0$ and σ=0 we start from a two-cluster anti-phase synchronized solution with clusters A and B of size $N_{A}$ and $N_{B}$ . Next we perform a stability analysis around the two-cluster solution. The Jacobian takes a block form with four blocks $J_{\theta \theta }$ , $J_{\theta k}$ , $J_{k\theta }$ , and $J_{kk}$ . For the phase-coupling block $J_{\theta k}$ it is easy to show that the matrix vanishes. Therefore the determinant of the entire Jacobian factorizes into those of the diagonal entities $\det J_{\theta \theta }\det J_{kk}$ , and the eigenvalues are the union of those of $J_{\theta \theta }$ and $J_{kk}$ . The eigenvalues of $J_{kk}$ turn out to be all negative. Thus the problem is reduced to determine the eigenvalues of $J_{\theta \theta }$ . At the two-cluster anti-phase equilibrium the couplings attain their equilibrium values ${\dot{k}}_{ij}=0$ , which leads to vanishing row sums and one zero eigenvalue, corresponding to the global phase shift of the two-cluster solution. Moreover, it can be shown that $\chi J_{\theta \theta }\chi =-J_{\theta \theta }$ with a flip matrix χ . Therefore the eigenvalues of $J_{\theta \theta }$ occur in symmetric pairs with m−1 positive values $\lambda_{k}=1-2k/N,\;k=1,2,\ldots ,m-1$ and m−1 negative values $\lambda_{-k}=-\left(1-2k/N\right),\;k=1,2,\ldots ,m-1$ for N=2m plus 2 zero eigenvalues; for N=2m+1 we have m positive and m negative values and one zero eigenvalue, $\lambda_{k},\lambda_{-k}$ as before with k=1,2,…m . This proves that with increasing system size N , we have an increasing number of unstable directions for the two anti-phase synchronized cluster solution.

What does this scaling mean in view of possible neural network applications? As stated in the introduction, individual Kuramoto oscillators are often used to model entire brain regions such as cortical areas, each treated as one oscillatory unit. For small system sizes such as N=10 or N=20 this could mean that one observes a pronounced metastability of synchronization patterns between brain areas. For a long dwell time they are synchronized in one pattern of two anti-phase synchronized clusters of areas, until suddenly one or a few of these areas switch their cluster affiliation: after the switch, they synchronize with the cluster complementary to the one they were synchronized with before the switch. The dwell time in one synchronization pattern decreases with the number of coupled areas, the more areas, the shorter the dwell time. We leave it open which assignment to a single Kuramoto oscillator is most appropriate, ranging from a single neuron to global areas in the brain.

### 3.5 Impact of disorder via noise or natural frequencies

**FIGURE 9:**
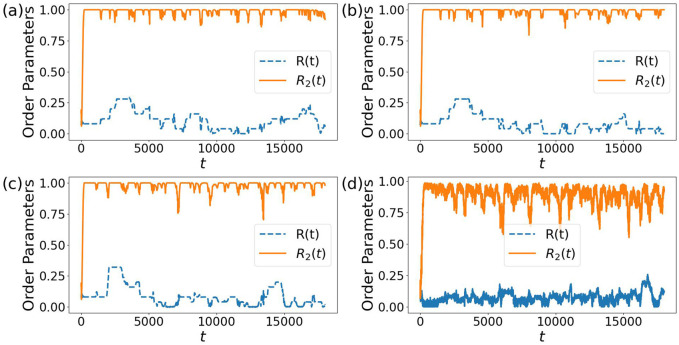
Time evolution of the order parameters R and $R_{2}$ for four noise strengths: **(a)** σ=0 , **(b)** σ=0.00001 , **(c)** σ=0.001 , **(d)** σ=0.1 , leading to incoherent oscillations underlying R and $R_{2}$ in **(d)**. Parameters N=50 , others as in Figure 2. We use the same random initial conditions to generate the four figures in panel **(a,d)**. For further comments see the main text.

In Kuramoto models with two non-reciprocally interacting populations, disorder in the frequencies or noise can stabilize a chiral phase, which is chiral in the sense that instead of static phase alignment a chiral motion is observed such that in spite of zero natural frequencies, oscillators spontaneously rotate either clockwise or anti-clockwise (Fruchart et al., 2021). In our version of the Kuramoto model with one population and adaptive non-reciprocal couplings to all other oscillators, the role of disorder or noise that we observed is toward an increase of escapes from a two anti-phase synchronized cluster state. Different strengths of noise still produce metastability in our model, as shown in Figures 9a–c. For the strongest noise amplitude in Figure 9d, the order parameter $R_{2}$ shows larger deviations from 1, indicating that temporarily several clusters have formed, but $R_{2}$ remains close to 1 and returns to this value again and again, so that remnants of the anti-phase synchronized two clusters are still visible. Since the phase difference between the two clusters is roughly π , noise is in general not sufficient for an escape where many oscillators overcome the distance and change their cluster affiliation at the same time, just a few succeed as the panels (c) in Figure 8 show. In particular, noise does not improve synchronization at an intermediate noise level; instead, due to a large phase difference, it monotonically weakens the perfect anti-phase cluster synchronization, allowing for longer escapes, and broadens a bit the in-phase synchronization within a cluster.

### 3.6 Effects of a sparse network topology

In the next version of the model, inspired by the Ref. (Kasatkin and Nekorkin, 2018), the dynamics of the oscillators and their adaptive couplings are modified to account for a connected but sparse random network topology. The coupling matrix $k_{ij}$ now evolves only along the links of a network represented by the adjacency matrix $A=\left[A_{ij}\right]$ . The phase dynamics are therefore weighted by the network structure:

$${\dot{\theta }}_{i}=\omega_{i}+\frac{1}{\sum_{j}A_{ij}}\overset{N}{\underset{j=1}{\sum }}A_{ij}k_{ij}\sin\left(\theta_{j}-\theta_{i}\right),$$ (3)

where $\sum_{j}A_{ij}$ is the degree of node i . The adaptive evolution of the coupling matrix is modified such that only links, which are already initially existing, update according to the asymmetric rules:

$${\dot{k}}_{ij}=\left\{\begin{array}{cc} -\varepsilon_{1}\left[k_{ij}+\sin\left(\theta_{i}-\theta_{j}-\frac{\pi }{2}\right)\right]A_{ij}, & i<j,\\ -\varepsilon_{2}\left[k_{ij}+\sin\left(\theta_{i}-\theta_{j}+\frac{\pi }{2}\right)\right]A_{ij}, & i>j,\\ 0, & i=j. \end{array}\right.$$ (4)

Using the dynamics (Equations 3, 4), we simulated networks of size N=10 over multiple realizations for varying average degrees ⟨k⟩ . The lifetime of the two-cluster anti-phase state was computed by monitoring the order parameters R and $R_{2}$ , identifying periods where $R<R_{\text{th}}$ while $R_{2}>R_{\text{th}}$ .

**FIGURE 10:**
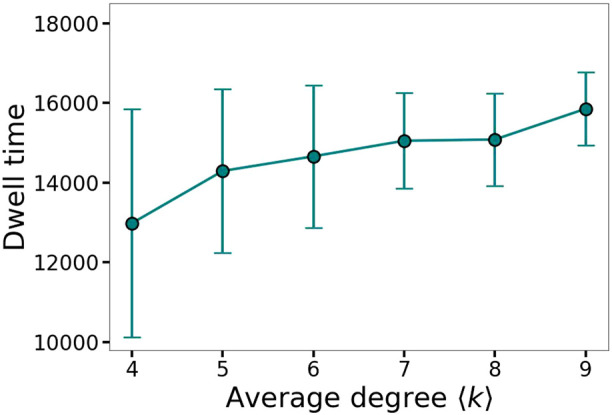
Increase of the dwell time in the vicinity of saddles with the network connectivity. The metastability is most pronounced for a fully connected network with average degree ⟨k⟩=9 and N=10 .

Figure 10 shows the mean and standard deviation of the two-cluster lifetimes over 100 realizations as a function of the average degree ⟨k⟩ . As ⟨k⟩ increases and the network becomes more densely connected, on average the dwell time increases and the metastability is more pronounced. The network topology introduces fluctuations in the dwell times across realizations, reflected in the error bars. Using our rough synchronization threshold of 0.8, we do not see a pronounced rearrangement of clusters of the synchronized oscillators, differently from (Kasatkin and Nekorkin, 2018), in our case the two anti-phase cluster configuration is quite dominant.

### 3.7 Kuramoto model with non-reciprocal and adaptive frequencies

Reciprocal synaptic plasticity alone can have the interesting effect that heterogeneous layered clusters with different frequencies emerge from homogeneous populations as the Fourier zero modes of the phase coupling function is included, leading to an additional constant term in the interaction part of the oscillators (Aoki, 2015). Here we implement a Kuramoto model, inspired by the framework outlined in (Aoki, 2015), but with asymmetric plasticity. In this model, the frequency of each oscillator, denoted by $\tilde{\omega_{i}}$ , depends on the coupling strengths, which evolve dynamically over time. The frequency of the i -th oscillator is given by the following equation:

$$\tilde{\omega_{i}}=\omega_{i}+\frac{\Gamma_{0}}{N}\overset{N}{\underset{j=1}{\sum }}k_{ij},$$

where $\Gamma_{0}\in \left[0,1\right]$ is a constant that influences the phase dynamics between oscillators. The phase evolution for each oscillator is governed by the equation:

$$\frac{d\theta_{i}}{dt}=\omega_{i}+\frac{1}{N}\overset{N}{\underset{j=1}{\sum }}k_{ij}\left(\Gamma_{0}-\sin\left(\theta_{i}-\theta_{j}+\alpha \right)\right),$$

where α is a constant that modulates the coupling strength between oscillators. The coupling matrix $k_{ij}$ now evolves according to the non-reciprocal plasticity rule as defined by Equation 2, with $\beta_{1}=-\frac{\pi }{2}$ and $\beta_{2}=+\frac{\pi }{2}$ . Initially, we set α=0 , which simplifies the model to the noiseless version of the proposed model (1) with $\Gamma_{0}=0$ .

Even when the initial natural frequencies $\omega_{i}$ are set to zero, a nonzero $\Gamma_{0}$ ensures that the frequencies $\tilde{\omega_{i}}$ remain time-dependent. This leads to incoherent oscillations among frequencies. Furthermore, when $\Gamma_{0}$ is nonzero, the phases $\theta_{i}$ of the oscillators also evolve, and again we observe metastability in the system. This metastable behavior persists for values of $\Gamma_{0}$ in the range [0,0.2] .

At small nonzero values of α∈[−0.04,0.04] , we observe the formation of two distinct clusters, with a phase difference of approximately π between them. As $\Gamma_{0}$ increases to the range (0.2,0.6) , the system still maintains two phase-locked clusters, but the phase difference between them is typically less than π . When $\Gamma_{0}$ exceeds 0.6 and lies in the range [0.6,1] , the oscillators become fully incoherent, and no phase locking is observed.

**FIGURE 11:**
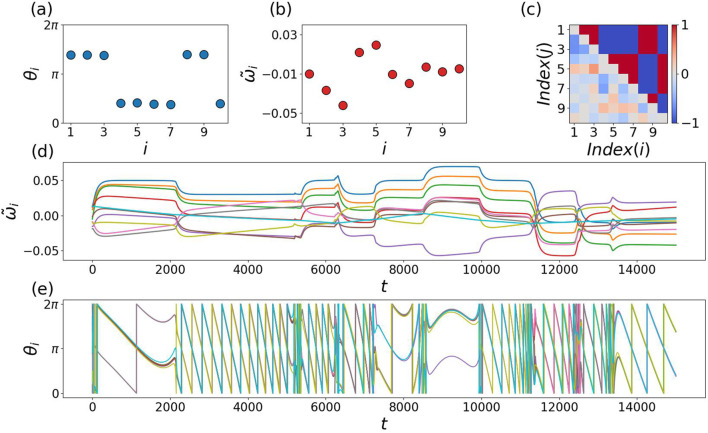
When frequencies become coupling dependent. Panel **(a)** shows a snapshot of the two anti-phase clusters of the oscillator phases. Panel **(b)** displays the instantaneous frequencies at the same snapshot time t = 15,000, revealing incoherent frequency dynamics. The evolution of the couplings is shown in panel **(c)**, where the upper couplings rapidly reach their equilibrium values of ± 1, while the lower couplings fail to do so. Panel **(d)** presents the time evolution of the frequencies over a longer time interval, and panel **(e)** shows the corresponding long-time evolution of the phases. Further details are discussed in the main text.

The time evolution of the order parameters and upper and lower couplings looks qualitatively the same as before. Whenever $R_{2}$ approaches 1 (not displayed in Figure 11), the oscillators’ phases naturally divide into two groups. Despite all oscillators starting with $\omega_{i}=0$ , they begin to oscillate due to the nonzero value of $\Gamma_{0}=0.1$ .

We show a snapshot at t=15000 of the phases and frequencies $\tilde{\omega_{i}}$ in Figure 11a,b, where each oscillator has a distinct $\tilde{\omega_{i}}$ that oscillates over time. The 10 oscillators are divided into two groups separated by a phase difference of π , although the frequencies are quite heterogeneous. In panel (Figure 11c), we display a snapshot of the coupling matrix $k_{ij}$ . The lower triangle of the matrix does not yet converge to its equilibrium values of ±1 due to the slow adaptation rate $\varepsilon_{2}=0.0001$ , the upper one does. One can easily read off which oscillators remain in the same cluster with upper couplings being attractive and which ones are in opposite clusters with upper couplings being repulsive. The long-time evolution of the phases $\theta_{i}$ (Figure 11e) indicates how the anti-phase synchronized clusters change together with the frequency patterns (Figure 11d) in time intervals between abrupt changes of the slope of ${\tilde{\omega }}_{i}$ and $\theta_{i}$ . Note that ${\tilde{\omega }}_{i}$ play the role of effective natural frequencies. Obviously they can considerably differ from the actual frequencies ${\dot{\theta }}_{i}$ , being larger in the time interval between t∈[2000,4000] than in the one between t∈[8000,10000] , although the opposite order is seen for ${\tilde{\omega }}_{i}$ .

## 4 Conclusions and outlook

We have studied generalized Kuramoto models of classical oscillators which have applications in particular to model oscillatory dynamics in neuronal networks. Strengths or weights assigned to synapses are in general not symmetric, therefore it is natural to model their dynamics via non-reciprocal couplings, where we have chosen Hebbian and anti-Hebbian rules for plasticity. Moreover, the time scales on which adaptation takes place need not be the same in both directions. For a certain range of parameters and in this combination of asymmetric coupling’s type and time-scale, we observe metastability of (in general) two anti-phase clusters of synchronized oscillators where a few oscillators (“switcher oscillators”) change their cluster affiliation. This is induced by a dynamical switch of the coupling type (attractive or repulsive) of connections ij to nodes j from switcher oscillators i , which happen on different time scales, depending on whether i<j or i>j . We see temporary remnants of dynamical realizations of Harary’s theorem, that is, the existence of two anti-phase synchronized clusters on an all-to-all topology, as long as the dynamics on the second time scale, parameterized by $1/\epsilon_{2}$ , can be neglected.

Regarding the system size, individual oscillators may represent mesoscopic neural populations such as cortical columns (Roberts et al., 2019). Therefore a small system size of N=10 or N=20 with pronounced metastability would include many neurons; here the increasing instability for large N , derived from our scaling analysis, would not apply to the individual neurons, but to a large number of brain areas. The ‘switcher’ mechanism would mean that some areas are flexible with respect to the choice of the cluster with which to synchronize. In view of experiments, first steps may amount to identify interactions which are Hebbian-like in one direction and anti-Hebbian-like in the opposite direction together with the time scales they are acting on.

In our current model with non-reciprocal phase shifts of ±π/2 , the system favors two anti-phase clusters. Different options exist to obtain states with more than two clusters or different collective behaviors (e.g., a splay state (Berner et al., 2021c; Singha and Gupte, 2016), or generalized chimeras (Parastesh et al., 2021; Khaleghi et al., 2019)). One would typically need to modify the interaction function to include higher-order interactions (Kundu and Ghosh, 2022) or alter the phase lag parameters (Kasatkin et al., 2017) away from strict orthogonality, as shown in previous studies on clustering in coupled oscillator networks (Okuda, 1993; Hansel et al., 1993). An open question is as to whether switchers between more than two clusters could be created due to the same kind of non-reciprocal adaptive couplings. Finally, in order to see new collective phenomena in neural networks, one may search for dynamical phases as result of non-reciprocal interactions in models of limit-cycle oscillators such as Stuart-Landau or Fitz-Hugh Nagumo oscillators, including amplitudes apart from oscillator phases as considered in this work.

## Data Availability

The original contributions presented in the study are included in the article/Supplementary Material, further inquiries can be directed to the corresponding author.
