# Supplementary material for: Metastability induced by non-reciprocal adaptive couplings in Kuramoto models
Source: Front Netw Physiol. 2026 Mar 26;6:1774273. doi: 10.3389/fnetp.2026.1774273 (PMC13061673; doi:10.3389/fnetp.2026.1774273)
Supplement: Supplementary file 1 [file DataSheet1.pdf]

Supplementary Appendix for “Metastability induced by non-reciprocal adaptive couplings in Kuramoto models”

Sayantan Nag Chowdhury [1], **and Hildegard Meyer-Ortmanns** [1*,2]

School of Science, Constructor University, Campus Ring 1, 28759 Bremen[1]
[2]*Complexity Science Hub, Metternichgasse 8, 1030 Vienna, Austria*

Correspondence*:
hmeyerortm@constructor.university

1 APPENDIX: STABILITY ANALYSIS OF TWO ANTI-PHASE CLUSTERS OF ARBITRARY SIZES

Consider the original fully connected Kuramoto model

$$\dot{\theta}_i = \frac{1}{N}\sum_{j=1}^{N} k_{ij}\sin(\theta_j - \theta_i),$$

in the absence of noise ($\sigma_{\text{noise}} = 0$) with $\omega_i = 0\ \forall\ i = 1, 2, 3, \cdots, N$. Assume the oscillators form two anti-phase clusters, named Cluster A and Cluster B, of sizes N_A and $N_B = N - N_A$, with phases

$$\theta_i = \begin{cases} \theta_A, & i \in \text{Cluster A}, \\ \theta_B = \theta_A + \pi, & i \in \text{Cluster B}. \end{cases}$$

The state vector is

$$\mathbf{x} = (\theta_1, \ldots, \theta_N, k_{ij} \text{ for } i \neq j) \in \mathbb{R}^{N+N(N-1)},$$

where we exclude the fixed diagonal elements $k_{ii} = 0$. Linearizing the dynamics around the two-cluster solution, the Jacobian takes the four-block form:

$$\mathbf{J} = \begin{pmatrix} \mathbf{J}_{\theta\theta} & \mathbf{J}_{\theta k} \\ \mathbf{J}_{k\theta} & \mathbf{J}_{kk} \end{pmatrix}.$$

with $\mathbf{J}_{\theta\theta}$: $N \times N$, phase–phase interactions; $\mathbf{J}_{\theta k}$: $N \times N(N-1)$, effect of couplings on phases; $\mathbf{J}_{k\theta}$: $N(N-1) \times N$, effect of phases on couplings; $\mathbf{J}_{kk}$: $N(N-1) \times N(N-1)$, coupling–coupling interactions. Let us denote the blocks of the Jacobian as follows:
Phase–Phase block: $\mathbf{J}_{\theta\theta}$ The entries are

$$(\mathbf{J}_{\theta\theta})_{ij} = \frac{\partial \dot{\theta}_i}{\partial \theta_j} = \frac{\partial}{\partial \theta_j}\left[\omega_i + \frac{1}{N}\sum_{m=1}^{N} k_{im}\sin(\theta_m - \theta_i)\right].$$

Evaluating the derivative gives

$$(\mathbf{J}_{\theta\theta})_{ij} = \begin{cases} -\frac{1}{N}\sum_{m\neq i} k_{im}\cos(\theta_m - \theta_i), & i = j, \\ \frac{1}{N}k_{ij}\cos(\theta_j - \theta_i), & i \neq j. \end{cases}$$

Phase–Coupling Block: $\mathbf{J}_{\theta k}$ This block captures how changes in couplings affect the phase dynamics:

$$(\mathbf{J}_{\theta k})_{i,(j\neq l)} = \frac{\partial \dot{\theta}_i}{\partial k_{jl}} = \begin{cases} \frac{1}{N}\sin(\theta_l - \theta_i), & j = i, \\ 0, & j \neq i. \end{cases}$$

Coupling–Phase Block: $\mathbf{J}_{k\theta}$ This block gives how the phases affect the evolution of couplings:

$$(\mathbf{J}_{k\theta})_{(i\neq j),m} = \frac{\partial \dot{k}_{ij}}{\partial \theta_m} = \begin{cases} -\varepsilon_1\cos(\theta_i - \theta_j - \pi/2), & i < j,\ m = i, \\ \varepsilon_1\cos(\theta_i - \theta_j - \pi/2), & i < j,\ m = j, \\ -\varepsilon_2\cos(\theta_i - \theta_j + \pi/2), & i > j,\ m = i, \\ \varepsilon_2\cos(\theta_i - \theta_j + \pi/2), & i > j,\ m = j, \\ 0, & \text{otherwise.} \end{cases}$$

Coupling–Coupling Block: $\mathbf{J}_{kk}$ Finally, the effect of couplings on themselves is diagonal:

$$(\mathbf{J}_{kk})_{(i\neq j),(l\neq m)} = \frac{\partial \dot{k}_{ij}}{\partial k_{lm}} = \begin{cases} -\varepsilon_1, & i < j \text{ and } (i,j) = (l,m), \\ -\varepsilon_2, & i > j \text{ and } (i,j) = (l,m), \\ 0, & \text{otherwise.} \end{cases}$$

Phase–Coupling Block is Zero For the two-cluster configuration, each oscillator in cluster A has phase θ_A, and each in cluster B has $\theta_B = \theta_A + \pi$. Thus, for any entry of the phase–coupling block

$$(\mathbf{J}_{\theta k})_{i,(j\neq l)} = \frac{\partial \dot{\theta}_i}{\partial k_{jl}} = \begin{cases} \frac{1}{N}\sin(\theta_l - \theta_i), & j = i, \\ 0, & j \neq i, \end{cases}$$

we have two possibilities:

- If θ_l and θ_i belong to the same cluster, then $\theta_l - \theta_i = 0 \implies \sin(\theta_l - \theta_i) = 0$.
- If θ_l and θ_i belong to different clusters, then $\theta_l - \theta_i = \pm\pi \implies \sin(\theta_l - \theta_i) = 0$ as well.

Hence, for all i, j, l,

$$(\mathbf{J}_{\theta k})_{i,(j\neq l)} = 0 \quad \Rightarrow \quad \mathbf{J}_{\theta k} = \mathbf{0}_{N\times N(N-1)}.$$

Eigenvalues via block-triangular structure For the two-cluster state, we have shown that the phase–coupling block vanishes:

$$\mathbf{J}_{\theta k} = \mathbf{0}_{N\times N(N-1)}.$$

Hence, the Jacobian becomes block lower-triangular:

$$\mathbf{J} = \begin{pmatrix} \mathbf{J}_{\theta\theta} & \mathbf{0} \\ \mathbf{J}_{k\theta} & \mathbf{J}_{kk} \end{pmatrix}.$$

Recall that the determinant of a block-triangular matrix is the product of the determinants of the diagonal blocks:

$$\det \begin{pmatrix} \mathbf{A} & \mathbf{0} \\ \mathbf{C} & \mathbf{B} \end{pmatrix} = \det(\mathbf{A}) \cdot \det(\mathbf{B}),$$

and applying this to $\mathbf{J}$ gives the characteristic polynomial:

$$\det(\mathbf{J} - \lambda \mathbf{I}) = \det(\mathbf{J}_{\theta\theta} - \lambda \mathbf{I}_N) \cdot \det(\mathbf{J}_{kk} - \lambda \mathbf{I}_{N(N-1)}).$$

By definition, the eigenvalues are the roots of $\det(\mathbf{J} - \lambda \mathbf{I}) = 0$. From the above factorization, the eigenvalues of $\mathbf{J}$ are exactly the eigenvalues of the two diagonal blocks:

$$\mathrm{eig}(\mathbf{J}) = \mathrm{eig}(\mathbf{J}_{\theta\theta}) \cup \mathrm{eig}(\mathbf{J}_{kk}).$$

This proves that, due to the vanishing phase–coupling block, the spectrum of the full Jacobian splits into the spectra of $\mathbf{J}_{\theta\theta}$ and $\mathbf{J}_{kk}$.
Reduction to the Phase–Phase Block From the previous discussion, we have

$$\mathbf{J}_{kk} = \mathrm{diag}(-\varepsilon_1, \dots, -\varepsilon_1, -\varepsilon_2, \dots, -\varepsilon_2),$$

where each diagonal entry is strictly negative. Therefore, all eigenvalues of $\mathbf{J}_{kk}$ satisfy $\lambda(\mathbf{J}_{kk}) < 0$. Since the full Jacobian $\mathbf{J}$ is block lower-triangular, $\mathrm{eig}(\mathbf{J}) = \mathrm{eig}(\mathbf{J}_{\theta\theta}) \cup \mathrm{eig}(\mathbf{J}_{kk})$, any positive eigenvalue of $\mathbf{J}$ can only arise from $\mathbf{J}_{\theta\theta}$. **Conclusion:** The stability of the two-cluster state is entirely determined by the $N \times N$ phase–phase block $\mathbf{J}_{\theta\theta}$. The $N(N-1) \times N(N-1)$ coupling–coupling block $\mathbf{J}_{kk}$ cannot generate any instability because it is diagonal with strictly negative entries. Hence, the analysis reduces to checking the eigenvalues of $\mathbf{J}_{\theta\theta}$ only, greatly simplifying the stability problem.

Properties of the Phase–Phase Block $\mathbf{J}_{\theta\theta}$ The entries of $\mathbf{J}_{\theta\theta}$ are real:

$$(\mathbf{J}_{\theta\theta})_{ij} = \begin{cases} -\frac{1}{N} \sum\limits_{m \neq i} k_{im} \cos(\theta_m - \theta_i), & i = j, \\ \frac{1}{N} k_{ij} \cos(\theta_j - \theta_i), & i \neq j. \end{cases}$$

Row sum and zero eigenvalue: Observe that for each row i, the sum of entries is

$$\sum_{j=1}^{N} (\mathbf{J}_{\theta\theta})_{ij} = -\frac{1}{N} \sum_{m \neq i} k_{im} \cos(\theta_m - \theta_i) \quad + \sum_{j \neq i} \frac{1}{N} k_{ij} \cos(\theta_j - \theta_i) = 0.$$

Therefore, $\mathbf{J}_{\theta\theta}$ always has one eigenvalue exactly equal to zero: $\lambda_0 = 0$. This zero eigenvalue corresponds to the rotational invariance of the Kuramoto model. Shifting all phases by a constant amount $\theta_i \mapsto \theta_i + \phi_0$ does not change the dynamics, so perturbations along this uniform phase shift direction neither grow nor decay. Hence, the zero eigenvalue represents a neutral mode associated with the global phase of the two-cluster solution.

Symmetry of eigenvalues. At the two-cluster anti-phase equilibrium, the couplings attain their asymptotic values:

$$k_{ij}^{\mathrm{eq}} = \begin{cases} -\sin(\theta_i - \theta_j - \pi/2), & i < j, \\ -\sin(\theta_i - \theta_j + \pi/2), & i > j, \end{cases}$$

where $k_{ii}^{\mathrm{eq}} = 0$. Using these equilibrium couplings, the entries of the phase–phase Jacobian become

$$(\mathbf{J}_{\theta\theta})_{ij} = \begin{cases} -\frac{1}{N} \sum\limits_{m \neq i} k_{im}^{\mathrm{eq}} \cos(\theta_m - \theta_i), & i = j, \\ \frac{1}{N} k_{ij}^{\mathrm{eq}} \cos(\theta_j - \theta_i), & i \neq j, \end{cases} \tag{1}$$

and one can verify that the row sums vanish: $\sum_{j=1}^{N} (\mathbf{J}_{\theta\theta})_{ij} = 0, \quad \forall i$. Hence, the Jacobian $\mathbf{J}_{\theta\theta}$ always has at least one zero eigenvalue, corresponding to the uniform phase shift mode: $\lambda_0 = 0$. All entries of $\mathbf{J}_{\theta\theta}$ are real. Let us define the exchange (or flip) matrix $\chi \in \mathbb{R}^{N \times N}$ as

$$\chi = \begin{pmatrix} 0 & 0 & \cdots & 0 & 1 \\ 0 & 0 & \cdots & 1 & 0 \\ \vdots & \vdots & \iddots & \vdots & \vdots \\ 0 & 1 & \cdots & 0 & 0 \\ 1 & 0 & \cdots & 0 & 0 \end{pmatrix}.$$

This matrix is self-inverse, $\chi^2 = I_N$, and it satisfies the relation $\chi \, \mathbf{J}_{\theta\theta} \, \chi = -\mathbf{J}_{\theta\theta}$. As a consequence, since $\chi \, \mathbf{J}_{\theta\theta} \, \chi = -\mathbf{J}_{\theta\theta}$ is a similarity transformation, the eigenvalues of $\mathbf{J}_{\theta\theta}$ occur in symmetric pairs: if λ is an eigenvalue, then $-\lambda$ is also an eigenvalue. This property is useful for analyzing the stability of the two-cluster solution.

Eigenvalue spectrum of $\mathbf{J}_{\theta\theta}$: The phase–phase block $\mathbf{J}_{\theta\theta}$ always has at least one zero eigenvalue corresponding to the uniform phase shift mode. Combining this with the eigenvalue symmetry under the exchange matrix χ, we can characterize the spectrum as follows:

- If $N = 2m+1$ is odd, $\mathbf{J}_{\theta\theta}$ has one zero eigenvalue, m positive eigenvalues, and m negative eigenvalues. Moreover, the positive and negative eigenvalues have equal absolute values.
- If $N = 2m$ is even, $\mathbf{J}_{\theta\theta}$ has two zero eigenvalues, $m - 1$ positive eigenvalues, and $m - 1$ negative eigenvalues, again with matching absolute values.

Hence, as N increases, the number of positive eigenvalues grows, indicating an increasing number of unstable directions for the two-cluster solution.

Eigenvalues of $\mathbf{J}_{\theta\theta}$: By explicitly solving $\mathbf{J}_{\theta\theta}$ for the two-cluster configuration, one finds that the positive eigenvalues take the form

$$\lambda_k = 1 - \frac{2k}{N}, \qquad k = 1, 2, \ldots, n_+,$$

where n_+ is the number of positive eigenvalues. The corresponding negative eigenvalues are

$$\lambda_{-k} = -\lambda_k = -\left(1 - \frac{2k}{N}\right), k = 1, 2, \ldots, n_-,$$

with $n_+ = n_-$, reflecting the eigenvalue symmetry of $\mathbf{J}_{\theta\theta}$. Thus, for general N, the eigenvalues of $\mathbf{J}_{\theta\theta}$ consist of symmetric positive and negative pairs, along with one or two zero eigenvalues depending on whether N is odd or even. As N increases, the number of positive eigenvalues increases, indicating that the two-cluster configuration becomes less stable in larger networks.

Numerical Validation: To investigate the local stability of the two anti-phase clusters, we analyze the phase-phase block of the Jacobian, $J_{\theta\theta}$ (given in Eq. (1)), corresponding to a given bipartition of N oscillators.

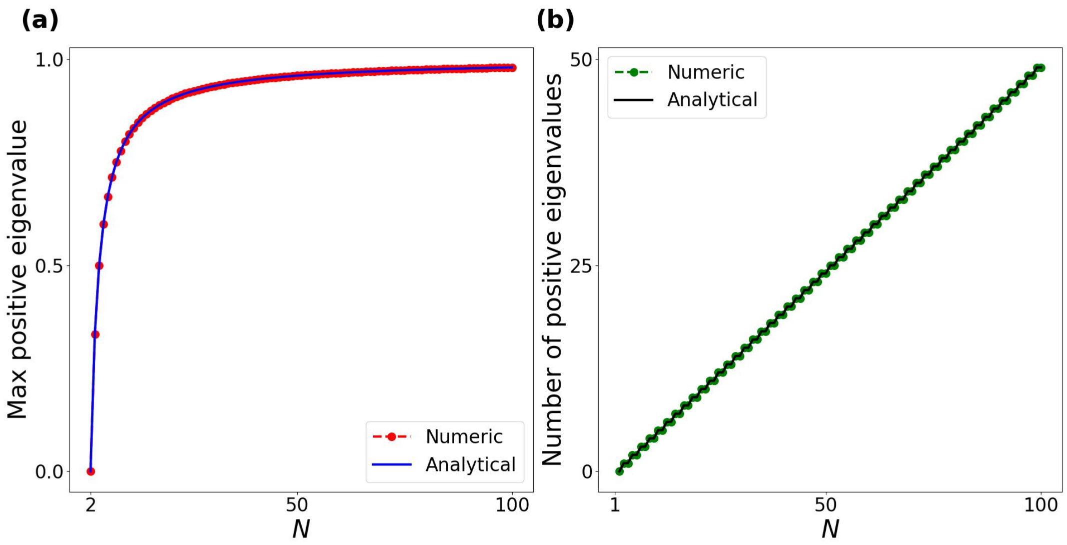

Figure 1. Stability analysis of the two-cluster anti-phase state using the phase-phase block $J_{\theta\theta}$ (given in Eq. (1)) of the Jacobian. **(a)** Maximum positive eigenvalue as a function of N. **(b)** Maximum number of positive eigenvalues as a function of the number of oscillators N. The results confirm that the numerical maxima agree with the analytical predictions.

For each bipartition, we construct the equilibrium phases $\theta_i = 0$ for one cluster and $\theta_i = \pi$ for the other, and compute the real parts of the eigenvalues of $J_{\theta\theta}$. Due to the large number of possible bipartitions, a full enumeration is computationally infeasible. Indeed, the total number of distinct bipartitions for a system of N oscillators is

$$\frac{1}{2}\sum_{k=1}^{N-1}\binom{N}{k} = 2^{N-1} - 1,$$

which for $N = 20$ amounts to $524{,}287$ possible configurations. Therefore, we adopt a random sampling strategy, selecting 500 random bipartitions for each N. This approach allows us to efficiently approximate the maximal number of positive eigenvalues and the largest positive eigenvalue while keeping the computational cost reasonable. The results of this procedure are summarized in Fig. (1), which shows the maximal positive eigenvalue and the number of positive eigenvalues as functions of N. Clearly, as N increases, both the number of positive eigenvalues and the maximum positive eigenvalue grow, with the latter approaching the theoretical limit of 1.
